# Clinical and Economic Assessment of MyDiaCare, Digital Tools Combined With Diabetes Nurse Educator Support, for Managing Diabetes in South Africa: Observational Multicenter, Retrospective Study Associated With a Budget Impact Model

**DOI:** 10.2196/35790

**Published:** 2023-08-07

**Authors:** Hemant Makan, Lindie Makan, Jacqueline Lubbe, Sarah Alami, Guila Lancman, Manuella Schaller, Cécile Delval, Adri Kok

**Affiliations:** 1 Centre for Diabetes Lenasia, Johannesburg South Africa; 2 Netcare Alberton Hospital Alberton South Africa; 3 Air Liquide Santé International Bagneux France

**Keywords:** diabetes mellitus, diabetes nurse educator, digital tool, MyDiaCare program, type 2

## Abstract

**Background:**

In South Africa, diabetes prevalence is expected to reach 5.4 million by 2030. In South Africa, diabetes-related complications severely impact not only patient health and quality of life but also the economy.

**Objective:**

The Diabetes Nurse Educator (DNE) study assessed the benefit of adding the MyDiaCare program to standard of care for managing patients with type 1 and type 2 diabetes in South Africa. An economic study was also performed to estimate the budget impact of adding MyDiaCare to standard of care for patients with type 2 diabetes older than 19 years treated in the South African private health care sector.

**Methods:**

The real-world DNE study was designed as an observational, retrospective, multicenter, single-group study. Eligible patients were older than 18 years and had at least 6 months of participation in the MyDiaCare program. The MyDiaCare program combines a patient mobile app and a health care professional platform with face-to-face visits with a DNE. The benefit of MyDiaCare was assessed by the changes in glycated hemoglobin (HbA_1c_) levels, the proportion of patients achieving clinical and biological targets, adherence to care plans, and satisfaction after 6 months of participating in the MyDiaCare program. A budget impact model was performed using data from the DNE study and another South African cohort of the DISCOVERY study to estimate the economic impact of MyDiaCare.

**Results:**

Between November 25, 2019, and June 30, 2020, a total of 117 patients (8 with type 1 diabetes and 109 with type 2 diabetes) were enrolled in 2 centers. After 6 months of MyDiaCare, a clinically relevant decrease in mean HbA_1c_ levels of 0.6% from 7.8% to 7.2% was observed. Furthermore, 54% (43/79) of patients reached or maintained their HbA_1c_ targets at 6 months. Most patients achieved their targets for blood pressure (53/79, 67% for systolic and 70/79, 89% for diastolic blood pressure) and lipid parameters (49/71, 69% for low-density-lipoprotein [LDL] cholesterol, 41/71, 58% for high-density-lipoprotein [HDL] cholesterol, and 59/71, 83% for total cholesterol), but fewer patients achieved their targets for triglycerides (32/70, 46%), waist circumference (12/68, 18%), and body weight (13/76, 17%). The mean overall adherence to the MyDiaCare care plan was 93%. Most patients (87/117, 74%) were satisfied with the MyDiaCare program. The net budget impact per patient with type 2 diabetes, older than 19 years, treated in the private sector using MyDiaCare was estimated to be approximately South African Rands (ZAR) 71,023 (US $4089) during the first year of introducing MyDiaCare.

**Conclusions:**

The results of using MyDiaCare program, which combines digital tools for patients and health care professionals with DNE support, suggest that it may be a clinically effective and cost-saving solution for diabetes management in the South African private health care sector.

## Introduction

### Background

The International Diabetes Federation estimated that in 2021, in South Africa, 4.2 million adults, aged between 20 and 79 years, will have diabetes, with almost 100,000 diabetes-related deaths annually [1]. The number of adults with diabetes is expected to reach 5.4 million by 2030.

There are several reasons for this increase in prevalence, mainly driven by type 2 diabetes. The risk of type 2 diabetes increases with age. However, the most important risk factor for diabetes is obesity [2]. In South Africa, the recent increases in urbanization and consumption of high-calorie diets, accompanied by diminished physical activity, have resulted in increased obesity: 69% of women and 39% of men are reported to be overweight or obese [2]. Obesity is estimated to account for 87% of type 2 diabetes cases in South Africa [2].

The South African health care system is already burdened by high rates of HIV infection, AIDS, tuberculosis, noncommunicable diseases, maternal and childhood mortality, and injury-related disorders. The predicted diabetes epidemic, with sequelae of microvascular and macrovascular complications, will further burden the already fragile health care sector and the South African economy [2]. In 2021, the total diabetes-related health expenditure in South Africa is estimated at US $7.2 billion [1]. Diabetes is known to have severely impacted the South African economy [3]. Indeed, about two-thirds of diabetes-related deaths occurred in working-class people younger than 60 years.

The occurrence of diabetes-related complications severely impacts patient health and quality of life, reduces productivity, and incurs high costs. The risk of developing micro- and macrovascular diabetes-related complications is known to increase with increasing glycated hemoglobin (HbA_1c_) levels. Microvascular complications increase above a threshold of 6.5%, and macrovascular complications increase above a threshold of 7% [4].

In South Africa, many people with diabetes do not receive optimal treatment [5-7]. A retrospective cross-sectional study reported that, in 2013, only 15.5% of people with type 2 diabetes reached the recommended target HbA_1c_ of less than 7% [8,9]. Furthermore, in the South African cohort of the International Diabetes Management Study, the 899 patients with type 1 or type 2 diabetes had a mean HbA_1c_ of 8.2%, with only 30% of patients reaching an HbA_1c_ below the 7% target [6]. The international real-world DISCOVER study program was a prospective observational study that assessed diabetes management in 15,992 patients with type 2 diabetes in 38 countries [10]. The South African cohort of the DISCOVER study had a mean HbA_1c_ of 9% at baseline, 8.2% after 6 months of follow-up, 8.4% after 12 months, and 8.3% after 24 months [5]. In comparison, in the global DISCOVER study, mean HbA_1c_ was 8.3% at baseline [11]. It is well established that suboptimal treatment increases the risk of diabetes complications [4], reduces quality of life [12], and increases the economic burden [13].

Digital tools, with or without support by a health care professional, have proven to be effective in lowering HbA_1c_ levels and promoting diabetes self-management [14-16]. These benefits have been confirmed in meta-analyses based on systematic reviews [14,15,17]. However, in South Africa, evidence to support the use of digital tools for diabetes management is scarce.

### Objectives

The real-world Diabetes Nurse Educator (DNE) study was designed to assess the benefit of the MyDiaCare program, combining digital tools (patient mobile apps and a health care professional platform) with DNE support, for diabetes management support in South Africa after at least 6 months of use. Furthermore, an economic study estimated the cost-effectiveness of MyDiaCare for patients with type 2 diabetes in the South African private sector.

## Methods

### Study Design

The real-world DNE study was designed as an observational, retrospective, multicenter, single-group study. The study was performed on South African patients with diabetes who had participated in the MyDiaCare program for at least 6 months.

### Program Description

**Figure 1:**
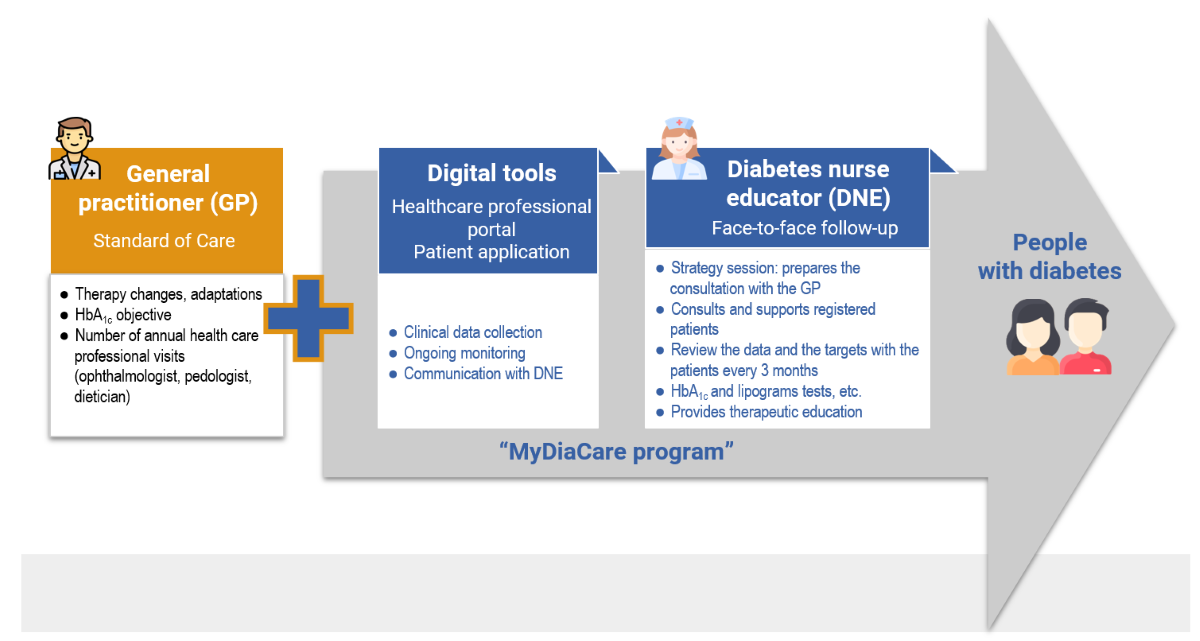
The MyDiaCare program. HbA_1c_: glycated hemoglobin.

The MyDiaCare program combines digital tools with regular DNE face-to-face interventions (See Figure 1).

### Patient Recruitment

Patients older than 18 years with type 1 or type 2 diabetes who had participated in MyDiaCare for at least 6 months were eligible for the study. Patients using another telemonitoring program, without complementary health insurance, or participating in another diabetes education program were ineligible. Furthermore, patients with limited or complete legal incapacity were not enrolled.

All eligible patients who presented at any one of the 2 participating health care centers for a routine visit between November 25, 2019, and June 30, 2020, and who agreed to participate in the study were enrolled. At enrollment, the patients performed the only study-specific visit.

### Study Procedures and Data Collected

During this study-specific visit, patient and diabetes-specific data were collected. In addition, relevant data collected in the MyDiaCare program were pseudonymized and extracted from the digital platform from the date the patient joined MyDiaCare until the date they left the program or at the latest on the June 30, 2020. These data included diabetes-related data (type of diabetes, date of diagnosis, diabetes treatment, concomitant treatments, diabetes complications, and comorbidities), clinical assessment (blood pressure, body weight, and waist circumference), and standard laboratory tests used for diabetes monitoring (HbA_1c_, low-density-lipoprotein [LDL] cholesterol, high-density-lipoprotein [HDL] cholesterol, total cholesterol, and triglyceride levels). Furthermore, patients’ diabetic care plans were assessed, including clinical targets (blood pressure, body weight, and waist circumference) and HbA_1c_ and blood lipid levels targets.

The BMI was calculated from the weight and height data collected. Moreover, patient, physician, and DNE satisfaction with MyDiaCare was assessed using self-administered and study-specific questionnaires.

### Study Outcomes

The outcome measures were the change in HbA_1c_ and cardiovascular risk factors (LDL, HDL, total cholesterol, triglyceride, systolic blood pressure [SBP], and diastolic blood pressure [DBP] levels, as well as body weight and waist circumference). Furthermore, the proportions of patients that achieved their targets for these parameters, the adherence to the care plans, and the satisfaction of the patients, physicians, and DNEs with MyDiaCare were also assessed. Adherence was defined as the percentage of tests or consultations performed over the tests planned, adjusted to the duration of follow-up for each patient. Patients’ satisfaction was assessed at least 6 months after joining the MyDiaCare program.

### Statistical Analysis

The data are described using descriptive statistics. Quantitative variables are presented as means and SD and medians with IQR. While qualitative variables are presented as frequencies with percentages. The numbers of missing data are indicated. Missing data were not replaced. Analyses were performed using SAS (version 9.4; SAS Institute).

### Economic Evaluation

#### Economic Study Objective

**Figure 2:**
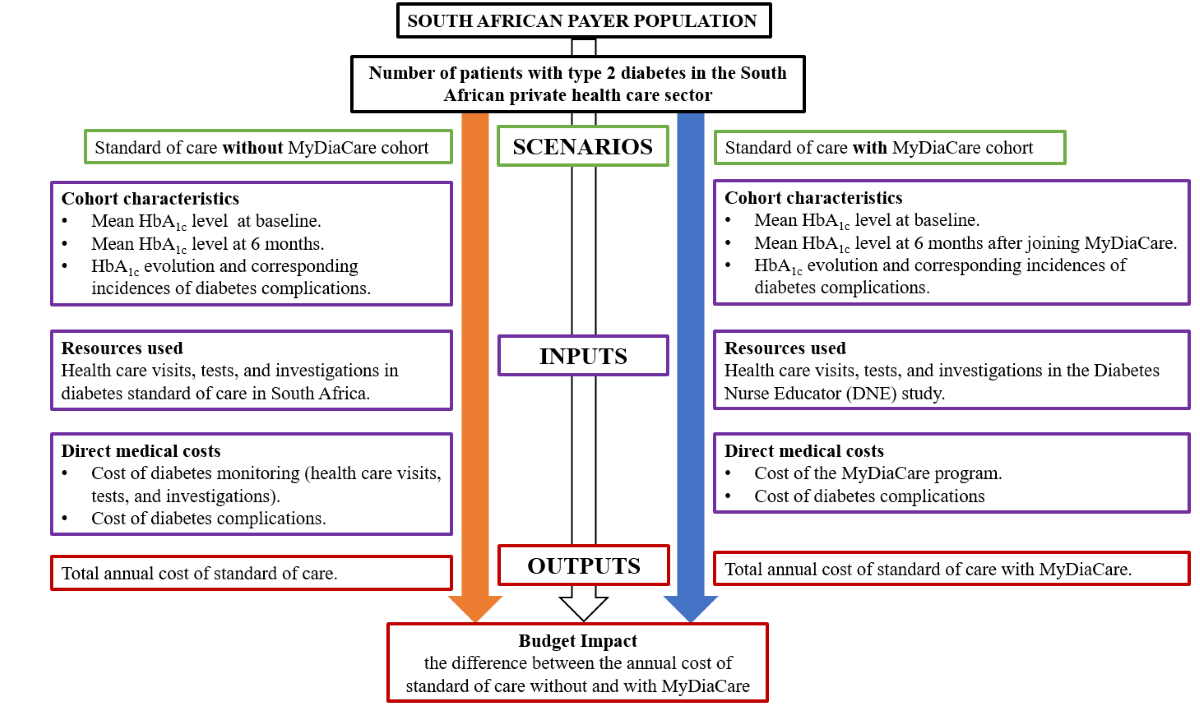
Economic study (budget impact analysis) methodology. HbA_1c_: glycated hemoglobin.

The economic evaluation focused on people with type 2 diabetes. A budget impact analysis was performed to estimate the economic impact of using MyDiaCare for treating patients with type 2 diabetes older than 19 years in the South African private health care sector. The model estimated the number of key medical events and budget impact of introducing MyDiaCare for managing adult patients with type 2 diabetes compared to standard of care. The analysis was performed according to HbA_1c_ improvement. The model compared 2 scenarios: type 2 diabetes management without MyDiaCare (standard of care) and management with MyDiaCare. The difference between the 2 scenarios provided an estimate of the number of diabetes complications avoided, the net budget impact per year, the cumulative budget impact over a 5-year time horizon, and the net budget impact per patient for the first year after introducing MyDiaCare. The methodology for the budget impact analysis is shown in Figure 2.

#### Simulated Populations

The model initially determined the mean HbA_1c_ level at baseline and then at 6 months for the 2 scenarios. The data from the DNE study, herein reported, were used to model type 2 diabetes management with MyDiaCare. The standard of care cohort was modeled using data from the South African cohort of the DISCOVER study [5,18].

The DISCOVER study was selected from a literature search that identified 6 publications that described type 2 diabetes management in South Africa [9,19-23]. The South African cohort of the DISCOVER study was chosen since it included patients from the private sector and had the necessary HbA_1c_ data to assess diabetes evolution [5]. Overall, the DISCOVER study was the most representative of the South African diabetes standard of care and was compared to the type 2 diabetes cohort of the DNE study.

#### Estimation of the Incidence of Diabetes Complications According to HbA_1c_ Levels

The increase in the number of diabetes complications according to HbA_1c_ levels was estimated using the reported annual incidence rates [5,21,24,25] and the increased risk according to HbA_1c_ levels reported in the ADVANCE study [4].

The annual incidence rates of macrovascular and microvascular events in South African patients with type 2 diabetes were obtained from the literature: 6% for myocardial infarctions [5], 4% for strokes [21], 17% for cardiovascular diseases [25], 25% for nephropathy [25], 15% for retinopathy [21], 13% for foot ulcers and diabetic foot [24], and 3% for amputations [25].

The international ADVANCE study assessed the association between HbA_1c_ levels in patients with type 2 diabetes and the risk of diabetes complications [4]. The study established HbA_1c_ thresholds for the occurrence of macrovascular and microvascular events. The risk of a macrovascular event increased by 38% for every 1% increase in HbA_1c_ level above the 7% threshold. While the risk of a microvascular event increased by 40% for every 1% increase in HbA_1c_ level above the 6.5% threshold. Below these thresholds, the risk of such events remained stable.

The incidence rates of diabetes complications were then adjusted according to HbA_1c_ levels at baseline and at 6 months [4]. The incidence of diabetes complications for the standard of care cohort was based on the mean HbA_1c_ level at baseline and after 6 months observed in the South African cohort of the DISCOVER study [5]. While for the MyDiaCare cohort, the HbA_1c_ levels observed in the DNE study were used.

The model initially determined the mean HbA_1c_ levels at baseline in the cohorts. The changes in HbA_1c_ levels from baseline to 6 months were then calculated. The model, for extrapolation over a 5-year time horizon, assumed that the HbA_1c_ levels beyond 6 months remained stable. The increased risk of diabetes complications, in increments of 0.1%, was estimated using the thresholds reported in the ADVANCE study [4]. In each cohort, the baseline incidences of the diabetes complications were then adjusted according to the increased risk to estimate the adjusted 6-month incidence rates for the complications.

#### Estimating the Diabetes Monitoring Frequency and Medical Resources Consumed in the Scenarios

In the standard of care scenario, the model includes health care visits and laboratory tests recommended for monitoring patients with type 2 diabetes by the Society for Endocrine, Metabolism, and Diabetes of South Africa (SEMDSA) guidelines published in 2017 [8].

The annual numbers of health care visits, as well as the numbers of HbA_1c_ and renal tests performed by patients with type 2 diabetes in the South African private sector, come from published real-life data [22]. These data were included in the model once verified and validated by the experts, selected as key opinion leaders in South Africa, involved in the primary market research (PMR).

In the MyDiaCare scenario, the medical resources used were:

1. The diabetes follow-up procedure in MyDiaCare:
  Laboratory tests: HbA_1c_, lipid tests, and microalbuminuria (MAU) (determined by albumin-to-creatinine ratio [ACR]).
  Health care visits: DNE, doctor, and dietetic consultations.
  Examinations: eye and foot screenings.
2. Concerning ophthalmologist and biokineticist visits; renal, creatinine, and potassium testing; and the urine dipstick, recommended by SEMDSA [8], but not systematically tracked in MyDiaCare, it was assumed that the program would not impact patients adherence. Consequently, the frequencies of these procedures in the standard of care cohort were also used for the MyDiaCare cohort.

#### The Cost of Medical Resources

The analysis incorporated direct medical costs associated with health care visits, tests, and investigations. The unit costs for the health care resources used (health care visits, tests, and investigations) were established from The National Health Reference Price List (NHRPL) [26], from the literature [13,27,28], and from interviews with the key opinion leaders. The unit costs were reported in different years. These costs were therefore inflated to 2021 values using the medical inflation rates from Statistics South Africa [29]. Similarly, the costs of diabetes complications were obtained from the literature and inflated [13,27,28].

The unit costs, adjusted for inflation, were multiplied by the frequencies of health care visits, tests, and investigations, as well as the incidences of diabetes complications, to calculate the total costs in each scenario (standard of care without and with MyDiaCare).

The cost of diabetes complications in each cohort was calculated based on the adjusted incidence and the unit cost of each complication. Cost of macrovascular and microvascular events were sourced from the literature. While the costs for foot ulcers and diabetic foot disease were estimated by key opinion leaders during PMR. The estimated cost of diabetes complications per event (in South African Rands [ZAR]) is shown in Table 1 with the source and cost inflated to 2021 values using the medical inflation rates from Statistics South Africa [30].

**Table 1:** Estimated costs of diabetes complication per event (in South African Rands). A currency exchange rate of South African Rand (ZAR) 1=US $0.053 is applicable.

Resource	Cost of event (ZAR)	Source	Cost in 2021 (ZAR)	

Myocardial infarction	43,415	Torborg et al [27]	65,025	
Stroke	34,722	Erzse et al [28]	38,245	
Cardiovascular disease	21,598	Erzse et al [28]	23,789	
Nephropathy	503,399	Erzse et al [28]	554,469	
Retinopathy	3973	Erzse et al [28]	4376	
Foot ulcers and diabetic foot	83,333	PMR^a^	83,333	
Amputations	323,418	Thompson et al [13]	339,589	

^a^PMR: Primary Market Research.

These calculations were used to estimate the number of diabetes complications avoided, the net budget impact per year, the cumulative budget impact over a 5-year time horizon, and the net budget impact per patient.

#### Sensitivity Analysis

One-way deterministic sensitivity analyses were performed. Analyses were performed on relevant parameters (eg, type 2 diabetes prevalence and mean HbA_1c_ levels). The individual parameters were varied over a range of plausible values (eg, low and high estimates) while holding other parameters constant to assess the effect on the overall outcome. If the optimal strategy did not change over the range of parameter values, then the model was considered to be insensitive to that parameter.

### Ethics Approval

The study was approved by the Research Ethics Committee of the South African Medical Association (registration number 1927/000136/08 NPC). All patients provided written informed consent before participating in the study. Furthermore, the study was conducted in compliance with Good Clinical Practice (International Council for Harmonization E6, the current version), the provisions of the Declaration of Helsinki (Fortaleza, Brazil, October 2013), and the applicable local regulatory requirements. The data was processed in accordance with the local regulations, notably the European Union Regulation 2016/679 (General Data Protection Regulation).

## Results

### Participant Characteristics

Overall, at 2 study centers, a total of 118 patients were included instead of the approximately 200 planned due to the COVID-19 pandemic. One patient, with a follow-up duration of only 3.45 months, was excluded from the analysis. In total, 117 patients were finally analyzed: 93% (109/117) with type 2 diabetes and 7% (8/117) with type 1 diabetes. Details concerning the demographic characteristics as well as diabetes treatments, complications, and comorbidities are summarized in Table 2.

**Table 2:** Participant characteristics at baseline.

Variable		Type 1 diabetes patients (n=8)		Type 2 diabetes patients (n=109)		Pooled (N=117)
**Age (in years)**						
	Median (IQR)	33.5 (29.5-35.5)	57 (51-64)		56 (49-63)	
	Mean (SD)	31.8 (6.5)	57.1 (10.8)		55.3 (12.3)	
Sex (female), n (%)		4 (50)		50 (46)		54 (46)
**Time interval between diabetes diagnosis and start of program (in years)**						
	Median (IQR)	11.2 (8.7-16.9)	9.8 (3.7-14.9)		9.9 (3.8-14.9)	
	Mean (SD)	14.3 (10)	10.5 (8.4)		10.7 (8.5)	
**HbA_1c_^a^ levels (%)**						
	Median (IQR)	7.9 (7.4-8.7)	7.7 (6.8-8.6)^b^		7.7 (6.8-8.6)^b^	
	Mean (SD)	8.2 (1)	7.8 (1.4)^b^		7.8 (1.4)^b^	
**Diabetes treatments, n (%)**						
	Oral therapy only	0 (0)	65 (60)		65 (56)	
	Insulin only	7 (88)	6 (6)		13 (11)	
	Insulin and oral therapy	1 (13)	38 (35)		39 (33)	
**Diabetes complications, n (%)**						
	Patients with complications	2 (25)	17 (16)		19 (16)	
	Peripheral neuropathy	0 (0.0)	11 (10)		11 (9)	
	Retinopathy	1 (13)	8 (7)		9 (8)	
	Nephropathy	1 (13)	1 (1)		2 (2)	
**Comorbidities, n (%)**						
	Patients with comorbidities	5 (63)	97 (89)		102 (87)	
	Dyslipidemia	5 (63)	90 (83)		95 (81)	
	Hypertension	4 (50)	71 (65)		75 (64)	
	Myocardial ischemia	0 (0)	10 (9)		10 (9)	
	Chronic kidney disease	0 (0)	2 (2)		2 (2)	
	Cardiac failure congestive	0 (0)	1 (1)		1 (1)	

^a^HbA_1c_: glycated hemoglobin.

^b^1 person with type 2 diabetes did not have a baseline HbA_1c_ level.

### Clinical Results

In the 79/117 patients with data at baseline and at 6 months, there was a clinically relevant mean decrease in HbA_1c_ levels of –0.6% (SD 1.3); median (IQR) –0.5 (–1.2 to 0.1); range (–3.5 to 3.5): from 7.8% at baseline to 7.2% at 6 months after starting MyDiaCare. These 79 patients comprised 8 patients with type 1 diabetes and 71 patients with type 2 diabetes. At 6 months, the mean HbA_1c_ level decreased from 8.2% to 7.4% in patients with type 1 diabetes and from 7.8% to 7.2% in those with type 2 diabetes.

**Figure 3:**
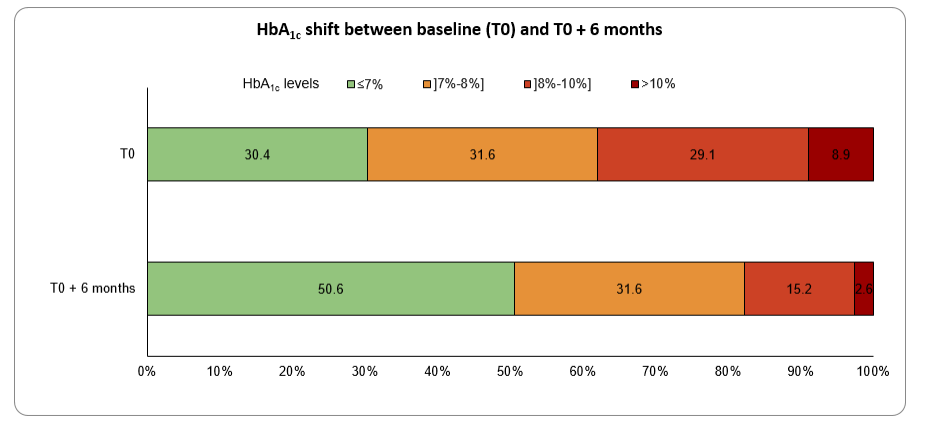
The proportion of patients with diabetes classified by glycated hemoglobin (HbA_1c_) levels at baseline and then at 6 months after starting the MyDiaCare program.

Among the 54 of 79 patients with a decrease in HbA_1c_ levels at 6 months, 80% (43/54) had a clinically relevant decrease of at least –0.4%. The clinically relevant decrease in the mean HbA_1c_ absolute change was also observed at time points ranging from 3 to 12 months. Moreover, the proportion of patients with HbA_1c_ levels <7% increased from 30.4% at baseline to 50.6% at 6 months (Figure 3).

**Figure 4:**
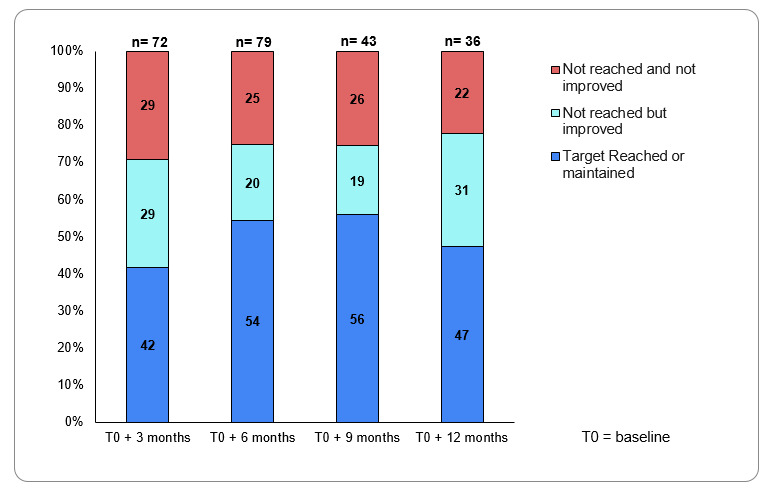
The proportion of patients with diabetes that reached their glycated hemoglobin (HbA_1c_) target at 3, 6, 9, and 12 months after starting the MyDiaCare program.

Most patients (43/79, 54%) reached or maintained their HbA_1c_ target levels and a further 20% (16/79) had improved HbA_1c_ levels but failed to reach their HbA_1c_ target (Figure 4).

At 12 months, there was a slight deterioration in the proportion of patients that reached their HbA_1c_ targets. However, only half of the patients had HbA_1c_ data at 12 months.

Concerning targets related to cardiovascular risk factors, more than 50% of patients reached or maintained their targets for the following parameters: 69% (49/71) for LDL cholesterol, 58% (41/71) for HDL cholesterol, 83% (59/71) for total cholesterol, 67% (53/79) for SBP, and 89% (70/79) for DBP. In contrast, less than 50% reached or maintained their targets: 46% (32/70) for triglycerides, 17% (13/76) for body weight, and 18% (12/68) for waist circumference. These data were not integrated in the economic evaluation.

Regarding adherence to the MyDiaCare care plan, a patient-specific schedule for laboratory tests: HbA_1c_, lipid tests, MAU (determined by ACR), for consultations: DNE, doctor, and dietetic consultations, and for examinations: eye and foot screenings. The mean overall adherence to the MyDiaCare care plan was 93%; 86% (100/117) of patients adhered to more than 70% of the scheduled tests and consultations in their care plan. The mean adherence to each test and consultation was between 98% and 100%, including physician and DNE consultations. However, for dietetic consultation and eye and foot examinations, the mean adherences were 59%, 57%, and 44%, respectively.

### The Levels of Satisfaction With MyDiaCare

From the patients’ perspective, most patients (87/117, 74%) were satisfied with their diabetes management, and 85% (99/117) agreed that MyDiaCare improved their diabetes management. The program allowed 62% (72/117) of patients to better understand their diabetes. Furthermore, most patients (105/117, 90%) noted that the presence of the DNE was useful or very useful. Finally, when patients completed the study-specific survey after participating for at least 6 months in the MyDiaCare program, 84% (98/117) wanted to continue using the program, and 89% (104/117) would recommend it to other patients.

All investigators reported that they were satisfied with the patients’ management, that they would use MyDiaCare long term, and that they would propose the program to at least 50% of their diabetes patients. They all felt that the program allowed them to better anticipate the patients’ complications and empowered their patients.

The 2 participating DNEs, 1 at each site, agreed that the program made them feel part of a medical team and allowed them to better anticipate patient complications. Furthermore, they found the tracking platform user-friendly, with the patients’ data easily available.

### Economic Evaluation

#### Simulated Populations

The base case analysis considered the economic impact if all patients with type 2 diabetes older than 19 years in the South African private sector joined the MyDiaCare program. The following annual prevalences (incidences) were used for the analysis: for year one, 328,118 patients (43,410), for year two, 368,295 (43,744), for year three, 408,454 (44,087), for year four, 448,603 (44,439), and for year five, 488,752 (44,800).

#### Overall Economic Impact of Diabetes Management With MyDiaCare

**Figure 5:**
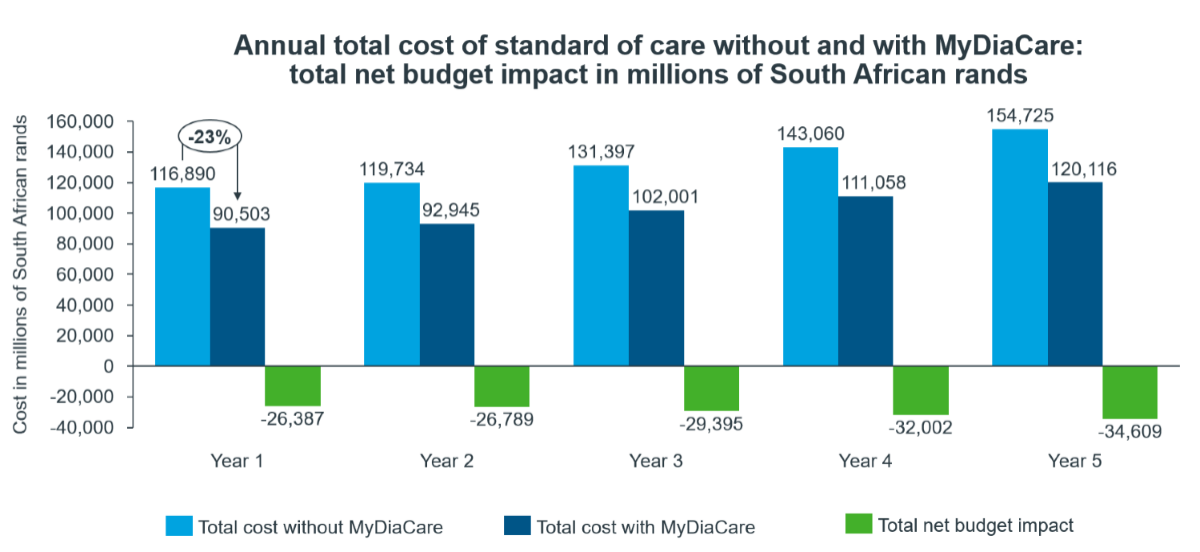
Estimated total annual costs of diabetes management in the scenarios without and with MyDiaCare (in millions of South African Rands).

The base case analysis considered the economic impact if all of the patients with type 2 diabetes treated in the South African private sector used the MyDiaCare program. Overall, the use of MyDiaCare to manage adults with type 2 diabetes in South Africa is estimated to lower the costs of diabetes management in South Africa in the first year by 26 billion ZAR (23%): from 117 billion ZAR with the current standard of care to 91 billion ZAR using MyDiaCare with standard of care (Figure 5). Please note that a currency exchange rate of ZAR 1=US $0.053 is applicable.

The net budget impact per patient with type 2 diabetes, older than 19 years, treated in the private sector using MyDiaCare was estimated to be approximately 71,000 ZAR (US $4089) during the first year of introducing MyDiaCare.

**Figure 6:**
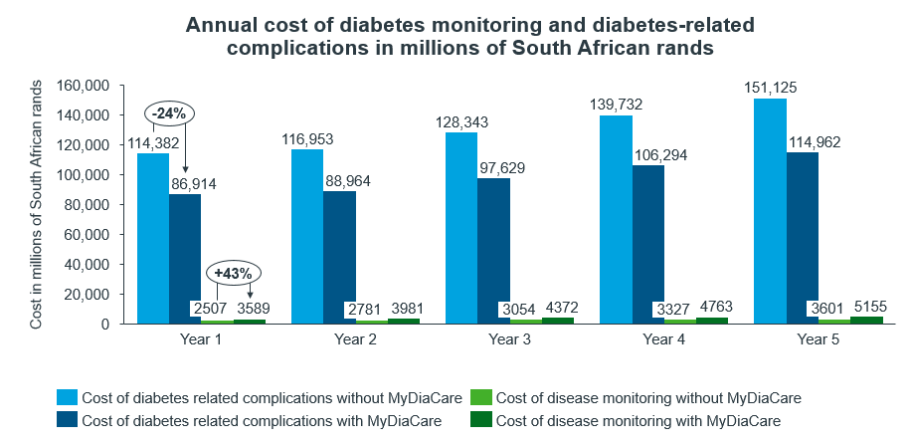
Estimated annual costs of diabetes-related complications and diabetes monitoring in the scenarios without and with MyDiaCare (in millions of South African Rands).

The use of MyDiaCare is estimated to increase the cost of diabetes monitoring (health care visits, tests, and investigations) during the first year by 1.1 billion ZAR (a 43% increase); see Figure 6. However, it substantially reduced the costs associated with diabetes complications by 27.5 billion ZAR (a 24% decrease), from 114 billion ZAR with current standard of care to 87 billion ZAR using standard of care with MyDiaCare.

The details of the results of the budget impact analyses are presented in the sections below.

#### Diabetes Complications Avoided and the Associated Costs

In the standard of care cohort, the mean HbA_1c_ level was 9% at baseline and 8.2% at 6 months. In the DNE study cohort with MyDiaCare, the mean HbA_1c_ was 7.8% at baseline and 7.2% at 6 months.

**Figure 7:**
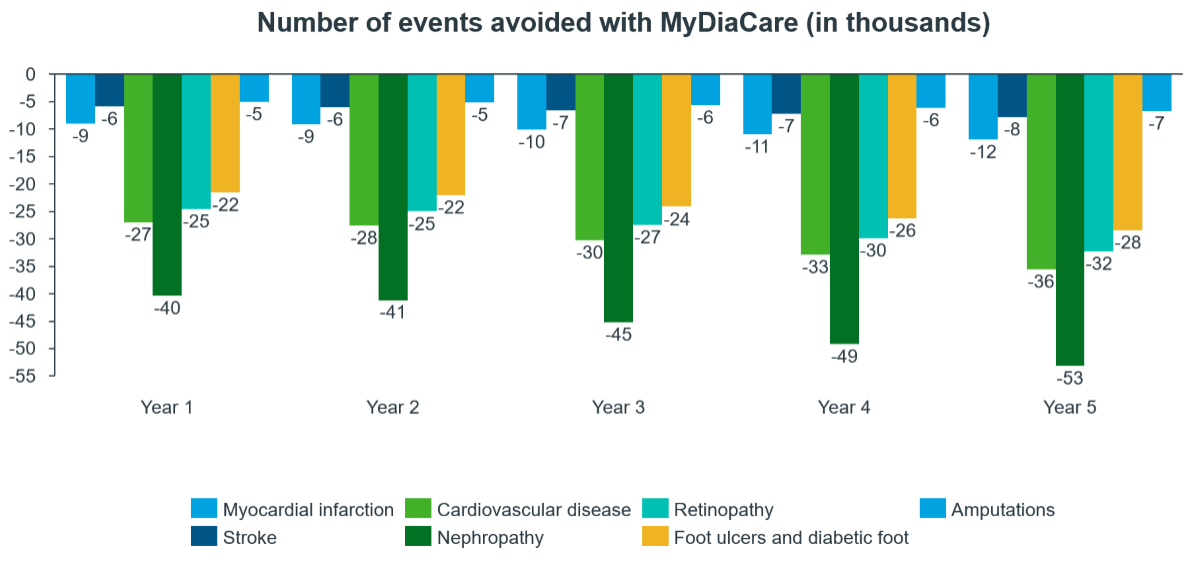
Estimated annual number of diabetes complications avoided with MyDiaCare (in thousands).

The estimated numbers of diabetes complications avoided (per year) in the scenario with MyDiaCare for type 2 diabetes patients in South Africa are shown in Figure 7.

**Figure 8:**
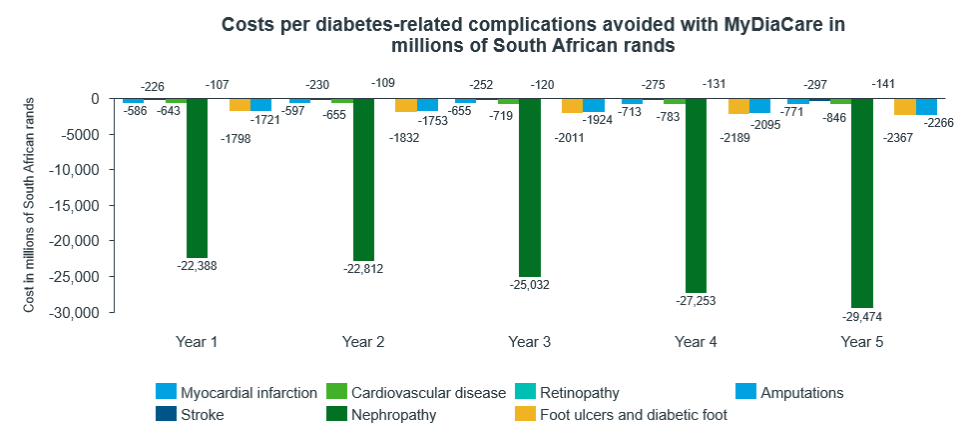
Estimated annual cost of diabetes complications avoided with MyDiaCare (in millions of South African Rands).

The costs associated with avoiding these complications are shown in Figure 8. The costs avoided are largely dominated by the nephropathy avoided, about 22 billion ZAR in the first year. Indeed, the incidence of nephropathy was higher in the type 2 diabetes cohort without MyDiaCare compared to the cohort with MyDiaCare.

#### Diabetes Monitoring Frequency and Cost Without and That With MyDiaCare

**Figure 9:**
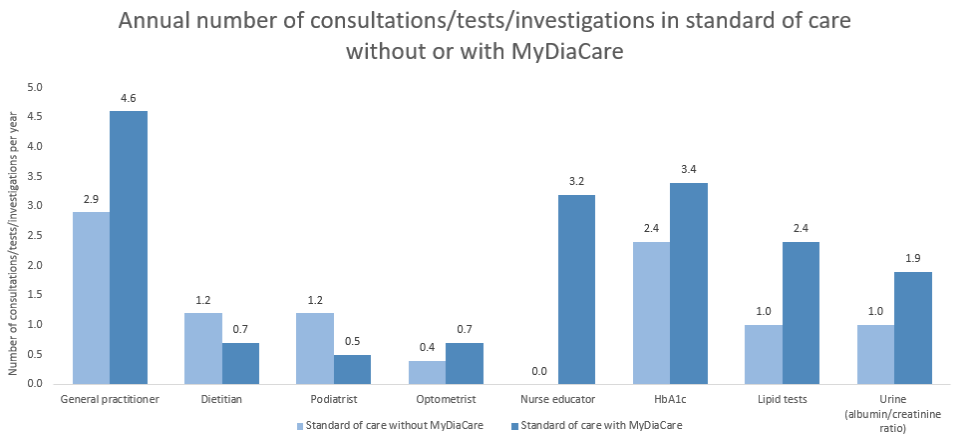
Comparison of the estimated number of health care visits, tests, and investigations required in the scenarios without and with MyDiaCare. HbA_1c_: glycated hemoglobin.

The use of MyDiaCare resulted in an increase in the annual number of visits with DNEs, general practitioners, and optometrists (Figure 9). Similarly, the annual number of HbA_1c_, lipids, and urine (albumin and creatine ratio) tests increased with MyDiaCare.

**Figure 10:**
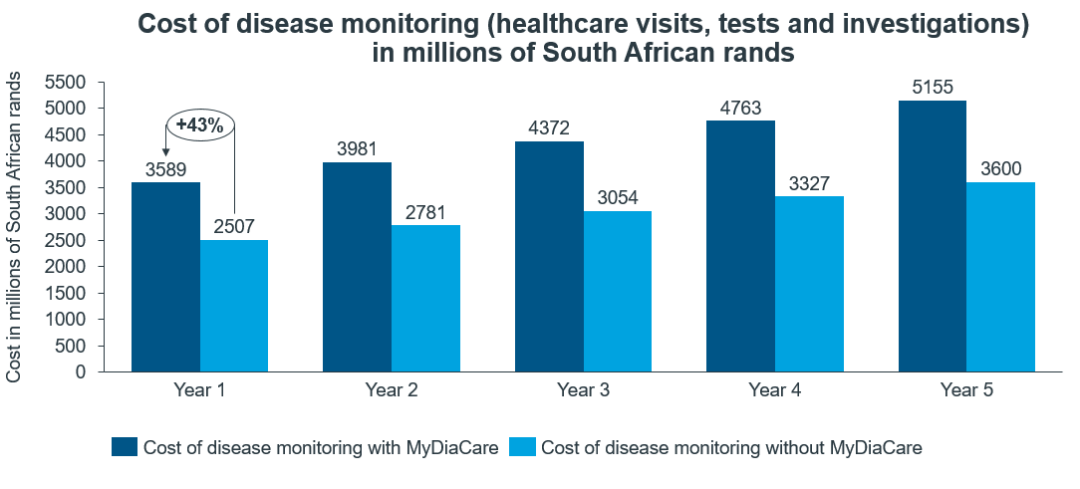
Estimated annual costs of diabetes monitoring (health care visits, tests, and investigations) in the scenarios with and without MyDiaCare (in millions of South African Rands).

The cost of diabetes monitoring (health care visits, tests, and investigations) in the first year was estimated to increase by 43% from 2.5 billion ZAR without MyDiaCare to 3.6 million ZAR with MyDiaCare (Figure 10).

#### Sensitivity Analysis

**Figure 11:**
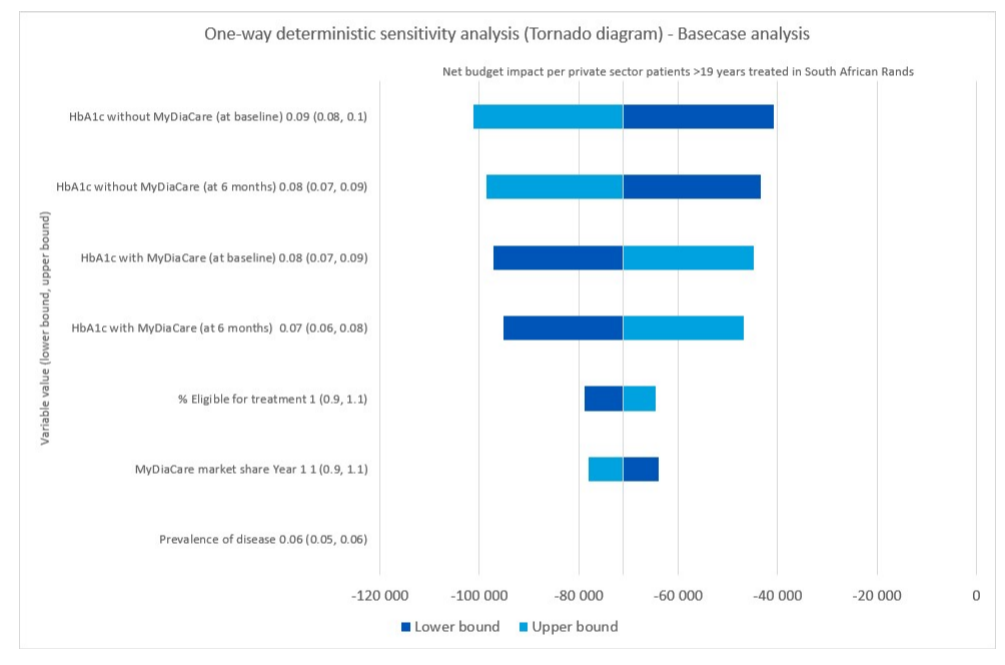
Results from the one-way deterministic sensitivity analysis: tornado plot showing the net change in budget per patient with type 2 diabetes, older than 19 years, with standard of care without and with MyDiaCare in South African Rands. HbA_1c_: glycated hemoglobin.

A 1-way deterministic sensitivity analysis was performed using the parameters that drive the budget impact model. The parameters were adjusted with a 10% variance. The tornado plot (Figure 11) below shows the change in the net budget per patient older than 19 years compared to the standard of care without and with MyDiaCare, as the base value for each parameter is adjusted according to the lower and upper bounds.

An additional scenario was run to address the differences observed in the MyDiaCare and standard-of-care groups. The scenario adjusted the mean baseline HbA_1c_ for the standard-of-care group to be equivalent to that of the MyDiaCare group at 7.8%. The scenario assumed that at 6 months, the mean HbA_1c_ levels in the standard-of-care group had not changed. Thus, the mean HbA_1c_ at 6 months remained at 7.8%. The mean HbA_1c_ levels in the MyDiaCare group were set at 7.8% at baseline and at 7.2% after 6 months to represent the impact of the DNE program and the intervention in the MyDiaCare cohort.

**Figure 12:**
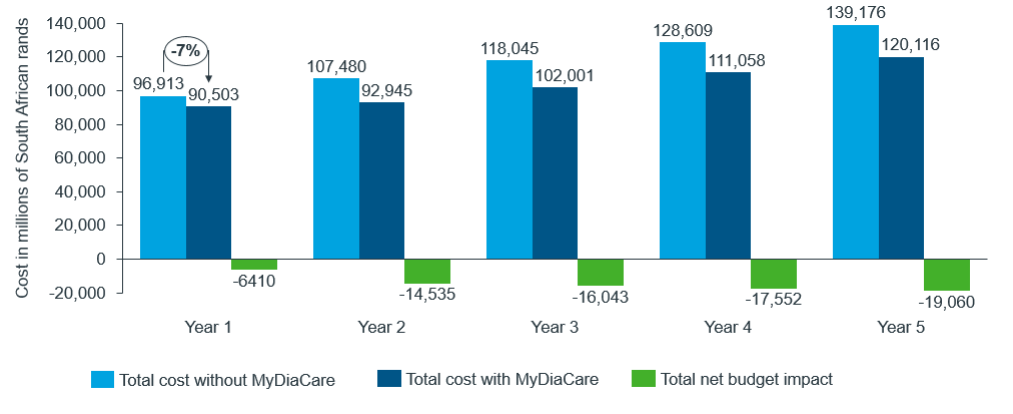
Results from a sensitivity analysis where the mean baseline HbA_1c_ levels in the groups were adjusted to equivalence: showing the net change in budget per patient with type 2 diabetes, older than 19 years, with standard of care without and with MyDiaCare in South African Rands.

The results on the total net budget comparing standard of care without and with MyDiaCare are shown in Figure 12. In this scenario, the cost of type 2 diabetes management in patients older than 19 years treated in the South African private health care sector is estimated to be reduced in the first year by 6410 million ZAR (365 million Euro)—a 7% difference in total cost between the 2 cohorts during the first year. The net budget impact in the first year per private sector patient older than 19 years treated is 17,652 ZAR (US $1016).

**Figure 13:**
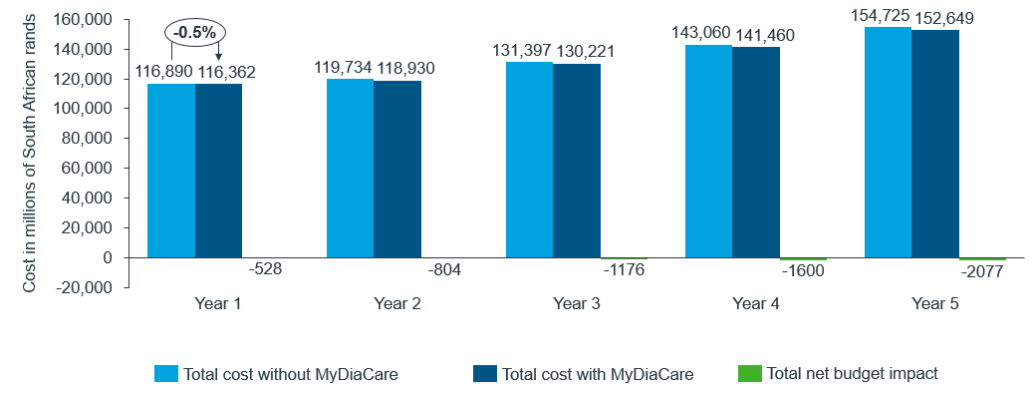
Total net budget impact comparing standard of care without and with MyDiaCare in the scenario that MyDiaCare has a market share of only 2% in the first year, growing at 1% per year over 5 years.

Furthermore, we investigated the scenario that MyDiaCare would only have a market share of 2% in the first year, growing at 1% per year over the 5-year time horizon (ie, reaching 6% at 5 years). The impact on the total net budget comparing standard of care without and with MyDiaCare is shown in Figure 13. In this scenario, the cost of type 2 diabetes management for patients older than 19 years treated in the South African private health care sector is estimated to be reduced by 528 million ZAR.

## Discussion

### Principal Results

In the DNE study, after 6 months of using the MyDiaCare program, the mean HbA_1c_ levels decreased by a meaningful –0.6%. from 7.8% at baseline to 7.2% at 6 months. This relevant decrease of –0.6% is substantial considering the low HbA_1c_ baseline level.

This decrease in mean HbA_1c_ levels is comparable to what has been reported in systematic reviews and meta-analyses after 3 to 10 months using mobile health interventions or telemedicine [14,15,17]. In the South African cohort of DISCOVER, the mean HbA_1c_ level was 9% at baseline and decreased to 8.2% after 6 months [5].

Several studies have investigated digital tools with limited or no intervention by a diabetes health care worker [16,31,32]. In South Africa, where health literacy is expected to be low, it is critical that digital tools be accompanied by regular health care visits, particularly DNE and physician visits. Indeed, a South African study conducted at a single institution found that knowledge concerning diabetic foot among patients with type 2 diabetes was suboptimal [24].

Regular and frequent visits with DNEs, in between visits with physicians, might play a role in decreasing HbA_1c_. The RENEWING HEALTH randomized controlled trial evaluated the benefit of a mobile health intervention with or without health counseling in patients with type 2 diabetes [31]. In the control group, there was only 1 telephonic counseling session by a DNE (4 months after baseline) compared to more frequent DNE face-to-face visits (every 3 months) in the health counseling group. In the Norwegian cohort of this trial, at 4 months, the HbA_1c_ levels were reduced on average by –0.23% with the mobile app, –0.41% with the mobile app and DNE counseling, and –0.39% with standard of care. These results are comparable with the –0.6% reduction in HbA_1c_ levels observed in this study.

Other studies that assessed mobile health interventions showed larger decreases in HbA_1c_ levels compared to this study. However, these larger decreases were to be expected since baseline HbA_1c_ levels were substantially higher than those in this study. After 3 months of using a mobile app with integrated coaching by a diabetes educator, the mean baseline HbA_1c_ level of 9.87% had decreased by –0.86% in patients that had completed the single-arm study and by –0.96% in those that actively used the mobile app and coaching. Furthermore, among active users, those with baseline HbA_1c_ levels ≥9% reduced these levels by on average –1.32% [32]. Similarly, after 4 months of using a digital tool comprising remote monitoring and lifestyle changes with coaching by a diabetes specialist, the mean HbA_1c_ level had significantly decreased by –0.8% from a baseline level of 8.9%. In patients with HbA_1c_ levels >9% at baseline, the HbA_1c_ decrease was even higher at –1.4% [16].

At 6 months, nearly 75% (59/79) of patients improved their HbA_1c_ levels: 54% (43/79) reached or maintained their HbA_1c_ targets, and a further 20% (16/79) improved their HbA_1c_ levels. Also, after 6 months of MyDiaCare, 50.6% (40/79) of patients had achieved the recommended HbA_1c_ level target of 7%, as compared to 30.4% (24/79) of patients at baseline. In comparison, after on average 2 years in the Central Chronic Medicine Dispensing and Distribution program, only 29.2% of patients had HbA_1c_ levels below 7% [7]. In the South African cohort of DISCOVER, the mean HbA_1c_ level was 9% at baseline and decreased to 8.2% after 6 months. Both the baseline HbA_1c_ levels (7.7%) and proportion of patients with HbA_1c_ levels below the targeted 7% at baseline (30.4%) and after 6 months (50.6%) in the MyDiaCare study were substantially better than those reported [5,7].

In addition to achieving HbA_1c_ targets, a large proportion of patients also achieved their targets for other cardiovascular risk factors, in particular LDL and HDL cholesterol and blood pressure levels.

It is noteworthy that both patients and health care professionals were satisfied with the MyDiaCare program. The MyDiaCare program provides innovative tools for patients and health care professionals. For patients, the mobile app allows them to self-manage their diabetes. While the health care platform allows physicians and DNEs to monitor diabetes evolution from a distance. In this program, the role of the DNE is critical: providing frequent and regular support for patients and physicians. Overall, MyDiaCare is a program designed to empower patients but also optimize diabetes management.

The real-world DNE study showed the benefit of MyDiaCare in improving HbA_1c_ levels. To provide further evidence of this metabolic benefit in the long term, we used HbA_1c_ as a proxy to estimate MyDiaCare’s impact in terms of diabetes complications and costs. Despite the estimated increased cost of diabetes management (health care visits, tests, and investigations), these costs were largely compensated for by the estimated costs avoided by reducing the number of diabetes complications (mainly nephropathy). The model estimated that in the private sector, about 26 million ZAR would be saved in the first year, with the cost of diabetes reduced by 23% compared to current standard of care. The annual cost savings for the private sector per patient during the first year were estimated to be 71,000 ZAR (US $4089). However, when we used the same mean baseline level of HbA_1c_ in the standard of care (results from a sensitivity analysis), the annual cost savings per patient during the first year were estimated to be 17,652 ZAR (US $1016). These savings are comparable with those previously reported [33,34]. For example, Smith and colleagues [33] evaluated the effectiveness and economic impact of a diabetes education program among adults with type 2 diabetes in South Texas. They found an HbA_1c_ decrease of –0.8% and a direct cost savings of US $2780 per year and per patient during the program. In another economic study, the annual savings per patient of an integrated care team model for targeted, high-risk Medicare patients with type 2 diabetes were estimated at US $5844 [34]. To our knowledge, no other reported economic study has estimated the cost of diabetes management in the South African private sector. This study suggests that increased diabetes monitoring, as observed in this study, may benefit not only patients and health care professionals but also private insurance.

### Limitations

The DNE study was designed to retrospectively collect real-world data concerning diabetes management in the South African private sector. The data collected therefore reflects current clinical practice. However, the retrospective design means that there are some missing data. In addition, the enrollment period of our study coincided with the COVID-19 pandemic, preventing us from recruiting the planned number of patients. Furthermore, there is no control group to evaluate the specific clinical effects that can be attributed to the MyDiaCare program. The DNE study was only performed at 2 health care centers, with mostly patients with type 2 diabetes enrolled. Thus, the results obtained may not be representative of diabetes management in the whole South African private health care sector. Baseline HbA_1c_ levels were lower than those already reported in other cohorts in South Africa. Moreover, 32% of patients with type 2 diabetes had reached their HbA_1c_ target level at baseline. Therefore, the DNE study population seems to correspond to a better-treated population than those reported, representing a selection bias [5-7]. Finally, the study was not designed to collect safety data, in particular cases of hypoglycemia and hyperglycemia.

The budget impact model has several limitations. First, the model is highly sensitive to HbA_1c_ levels at baseline and at 6 months, since these levels were used to estimate the incidences of diabetes-related complications where the main cost benefits were observed. Indeed, mean baseline HbA_1c_ levels in the standard of care cohort without MyDiaCare were higher than in the cohort with MyDiaCare. This difference would have impacted the estimated numbers of diabetes-related complications in these cohorts and the overall economic impact. Second, the HbA_1c_ levels in the standard of care cohort were estimated using the South African cohort of DISCOVER, but these data may not be representative of all patients and centers in South Africa [5]. Third, in the budget impact model, the HbA_1c_ levels obtained at 6 months were kept constant for the remainder of the model time horizon, and the method used to estimate long-term complications was the same for the first year and for subsequent years. The maintenance of HbA_1c_ levels after 6 months for the model is a hypothesis based on the continual effectiveness of MyDiaCare program to maintain these levels. Finally, the legacy effect, which is the continual long-term increase in risk despite improved glycemic control, and the difference in cumulative glycemic exposure, estimated by HbA_1c_, were also not considered in the economic model.

### Conclusions

Diabetes is a major public health challenge in South Africa. The increase in prevalence and related severe complications is expected to impact not only patient health and quality of life but also productivity and the South African economy. Many figures confirm that diabetes management in South African patients is, at present, suboptimal, with low percentages of patients reaching their targets for HbA_1c_ and other cardiovascular risk factors and adhering to guidelines. The MyDiaCare program, which combines digital tools for patients and health care professionals with DNE support, may be a clinically effective and cost-saving solution for diabetes management in the South African private health care sector. These results need to be confirmed in further studies.

## Abbreviations

ACR: albumin-to-creatinine ratio
DBP: diastolic blood pressure
DNE: Diabetes Nurse Educator
HbA_1c_: glycated hemoglobin
HDL: high-density lipoprotein
LDL: low-density lipoprotein
MAU: microalbuminuria
NHRPL: National Health Reference Price List
PMR: primary market research
SBP: systolic blood pressure
SEMDSA: Society for Endocrine, Metabolism, and Diabetes of South Africa
